# Copying Ink without a Press

**Published:** 1882-03

**Authors:** 


### COPYING INK WITHOUT A PRESS.

A paper on this subject by Prof. Attfield, F. R. S., etc., was lately read at the annual Pharmaceutical Conference at York. The author stated that for the past thirteen years all letters, reports, etc., that he had written had been transcribed into an ordinary thin paper copying book with no more effort than was employed in using a piece of blotting-paper. It had only been necessary to place the page of writing, note size, letter size, or even foolscap, in the letter-book, and use a leaf of the letter-book just as one would use a leaf of blotting paper. The superfluous ink that would go into blotting paper went on to the leaf of the letter-book, and, showing through the thin paper as usual, gave, on the other side of the leaf, a perfect transcript of the letter. Any excess of ink on the page either of the letter or of the copying-paper was removed by placing a sheet of blotting-paper between them and running one's hand firmly over the whole in the ordinary manner.

This ready transcription was accomplished, as would be anticipated, by using ink which dried slowly. Indeed, obviously, the ink must dry slowly sufficiently for the characters at the top of a page of writing to remain wet when the last line was written. While it must dry sufficiently fast to preclude any chance of the copied page being smeared while subsequent pages were being covered. The drying must also be sufficiently rapid to prevent the characters "setting off," as printers term it, from one page on to another after folding.

The author then alluded to some difficulties attending the employment of the ink which had prevented its becoming an article of wholesale trade, but, he said, any chemist and druggist could make it and sell it, giving directions for use to customers. He himself had used it from year's end to year's end without any trouble whatever. It would be particularly useful to professional men and private persons.

The principle of the method of preparation consisted in dissolving a moderately powerful hygroscopic substance in any ordinary ink. After experimenting on all such substances known to hin, he gave the preference to glycerin. Reduce, by evaporation, ten volumes of ink to six, then add four volumes of glycerin. Or manufacture some ink of nearly double strength and add to any quantity of it nearly an equal volume of glycerin.—*Scientific American.*

